# Nanofibrillated Cellulose Surface Modification: A Review

**DOI:** 10.3390/ma6051745

**Published:** 2013-05-03

**Authors:** Karim Missoum, Mohamed Naceur Belgacem, Julien Bras

**Affiliations:** Laboratoire de Génie des Procédés Papetiers (LGP2)—Laboratory of Pulp, Paper and Graphic arts sciences, UMR CNRS 5518, Grenoble INP-Pagora-461, rue de la papeterie, 38402 Saint-Martin-d’Hères, France; E-Mails: karim.missoum@lgp2.grenoble-inp.fr (K.M.); naceur.belgacem@grenoble-inp.fr (M.N.B.)

**Keywords:** nanofibrillated cellulose (NFC), physical adsorption, chemical surface modification, polymer grafting

## Abstract

Interest in nanofibrillated cellulose (NFC) has increased notably over recent decades. This bio-based nanomaterial has been used essentially in bionanocomposites or in paper thanks to its high mechanical reinforcement ability or barrier property respectively. Its nano-scale dimensions and its capacity to form a strong entangled nanoporous network have encouraged the emergence of new high-value applications. It is worth noting that chemical surface modification of this material can be a key factor to achieve a better compatibility with matrices. In order to increase the compatibility in different matrices or to add new functions, surface chemical modification of NFC appears to be the prior choice to conserve its intrinsic nanofibre properties. In this review, the authors have proposed for the first time an overview of all chemical grafting strategies used to date on nanofibrillated cellulose with focus on surface modification such as physical adsorption, molecular grafting or polymer grafting.

## 1. Introduction

Cellulosic materials have been thoroughly investigated and reviewed over recent decades as regards their surface modifications and applications [1,2,3]. Arising from wood and more especially from cellulose, there has been a growing interest for several years for bio-nanoparticles produced from cellulosic sources. Two main families have been identified: (i) NCC (NanoCrystalline Cellulose) and (ii) NFC (Nanofibrillated Cellulose). The present review focuses on NFC. The different pretreatments and devices available for production are presented and focus is proposed on the chemical surface modifications performed.

Nanofibrillated Cellulose (NFC) refers to cellulose fibers that have been fibrillated to achieve agglomerates of cellulose microfibril units; NFCs have nanoscale (less than 100 nm) diameter and typical length of several micrometers. Several denominations exist for describing such material and most often Nano/MicroFibrillated Cellulose (NFC/MFC) is used. An ISO standard group is working on an international denomination and first results were considered during the last Technical Association of the Pulp and Paper Industry (TAPPI) conference 2012 in Montreal (Canada) [4,5]. The authors will keep the NFC denomination throughout the manuscript.

Nanofibrillated cellulose is described as a long and flexible cellulosic nano-material and is obtained from cellulose fiber by mechanical disintegration. Several methods are currently used and are detailed latter. The first successful isolation of cellulose microfibrils was reported 30 years ago by Turbak *et al.* and Herrick *et al.* [6,7] using a Gaulin laboratory homogenizer but it was only 20 later that uses for these materials first appeared. Dilute suspensions of cut cellulose fibers from softwood pulp were treated by high shear forces to yield individualized cellulose microfibrils. The resulting suspensions showed a clear increase in viscosity after several passes through the homogenizer. Indeed, NFCs tend to form an aqueous gel at very low concentration (2% wt.) due to the strong increase of specific surface area and consequently the higher number of hydrogen bonds (arising from surface hydroxyl groups) for the same volume in comparison to native cellulosic fibers. Since the 1980s, other mechanical treatments have been performed and different raw materials, pre-treatments or post treatments have been tested. This is detailed in the following sections.

Nanofibrillated cellulose displays two main drawbacks, which are associated with its intrinsic physical properties. The first one is the high number of hydroxyl groups, which lead to strong hydrogen interactions between two nanofibrils and to the gel-like structure once produced. The second drawback is the high hydrophilicity of this material, which limits its uses in several applications such as in paper coating (increase of dewatering effect) or composites (tendency to form agglomerates in petro-chemical polymers). The most feasible solution to this is chemical surface modification to reduce the number of hydroxyl interactions and also to increase the compatibility with several matrices. This review details firstly the different devices and pretreatments to produce NFC followed by the surface modification of this material. To the best of our knowledge, no reviews in this field have been conducted in order to sum-up all the strategies available.

## 2. NFC Process Manufacturing

### 2.1. Sources

Nanofibrillated cellulose is currently manufactured from a number of different cellulosic sources. Wood is obviously the most important industrial source of cellulosic fibers, and is thus the main raw material used to produce NFC. Bleached Kraft pulp is most often used as a starting material for NFC production [8,9,10,11,12], followed by bleached sulfite pulp [13,14,15].

**Figure 1:**
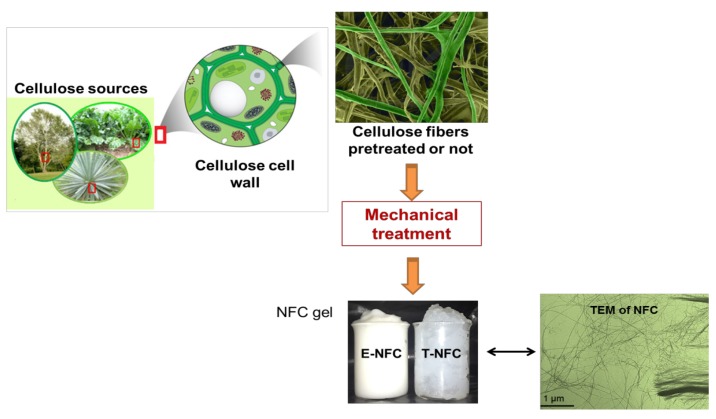
Description of classical process to obtain nanofibrillated cellulose (NFC). Different cellulose sources (wood or annual plant) followed by extraction of cellulose fibers from the cell wall, using different mechanical treatments, thus yielding a NFC gel suspension.

Whatever the source used, Figure 1 illustrates the general process used and the principle required to produce NFCs.

In the literature, diverse non-wood sources have already been used to produce NFC. For example, it can be extracted from sugar beet pulp [16,17], wheat straw and soy hulls [18], sisal [19], bagasse [20], palm trees [21], ramie, carrots [22], luffa cylindrica [23], *etc.*

Up to date, it seems that, contrary to Nanocrystalline Cellulose (NCC), the raw materials have only a little influence on the final NFC properties, even though they play a significant role on the processing energy consumption. Very recently, Rodionova *et al.* [24] analyzed more in detail two different bleached pulps (Norway Spruce and Eucalyptus pulps) for specifically pretreated (2,2,6,6-Tetramethyl-piperidin-1-oxyl commonly abbreviated as TEMPO pretreatment) NFC. The authors claimed that the self-standing films made from oxidized Norway spruce showed better transparency, visual appearance and tensile strength compared to Eucalyptus pulp.

### 2.2. Devices

**Figure 2:**
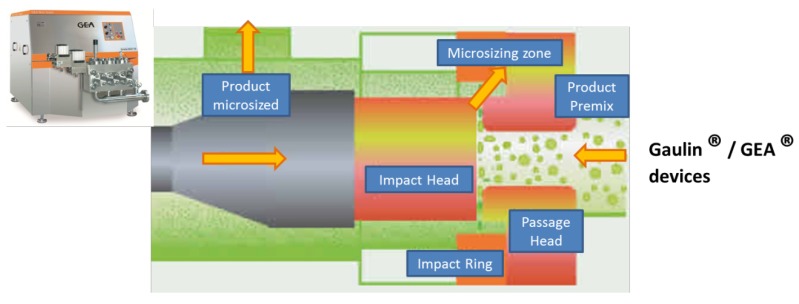
Description of homogenizer systems commonly used for NFC production—Gaulin and GEA systems.

**Figure 3:**
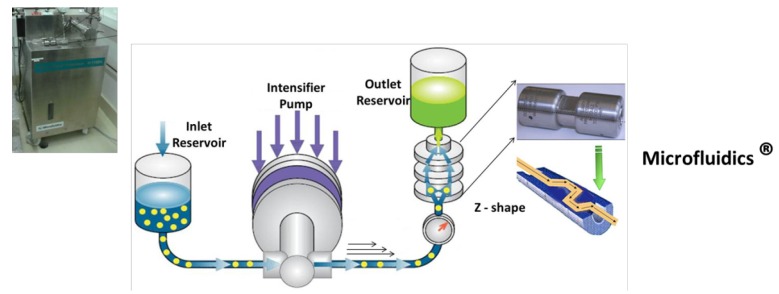
Description of one of most used devices to produce NFC—the microfluidizer from Microfludics ^®^.

**Figure 4:**
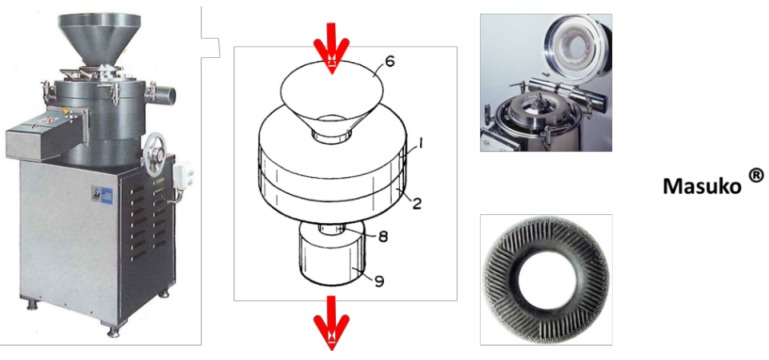
Grinder system developed by Masuko ^®^ for the fibrillation of fiber slurry in order to obtain an NFC suspension.

NFC is manufactured from a pulp suspension mainly using a mechanical treatment (Figure 2). Since the first production of nanofibrillated cellulose in the 80s, several methods have been developed to increase the production yield and the quality of the NFC. Up to now, three main families of devices have been used for the production of NFC, as presented in Figure 2, Figure 3 and Figure 4. The (i) homogenizer system grouping Gaulin^®^ homogenizer machine (from APV a SPX Inc. brand, West Sussex, UK) or the GEA^®^ homogenizer (sold by Niro Soavi, Parma, Italy) (Figure 2); (ii) the Microfludizer^®^ (developed by Microfludics Inc., Newton, MA, USA) (Figure 3); and (iii) the grinder devices like Masuko^®^ systems (Figure 4). Each Figure is presented and the corresponding device is described.

All the first three machines are based on a high pressure homogenizing system. From a cellulose fiber suspension, the slurry is pumped through valves and injected into pistons (for Gaulin^®^ and GEA^®^) or a high pressure chamber fibrillation (for microfluidizer^®^) around 8000 psi (55 MPa), 17,000–22,000 psi (120–150 MPa) and 10,000–30,000 psi (70–210 MPa) for Gaulin^®^, GEA^®^ and Microfluidizer^®^ machines respectively.

(i) For the homogenizer systems (Figure 2) (*i.e.*, Gaulin and GEA devices), the strong mechanical shearing, combined with the high pressure, initiates the fibrillation of fibers.

The non-homogenized product enters into the valve area at high pressure and low velocity. The pressure is increased when pressure is applied by the pneumatic valve shaft, closing the adjustable gap between the impact head and the passage head. The homogenizing effect is caused by the product entering the valve inlet at pressure. As it passes through the minute gap, the velocity quickly increases while the pressure rapidly decreases to atmospheric pressure. The homogenized product impinges on the impact ring and exits at a sufficient pressure for moving to the next processing stage. The ensuing fibrillated fibers are cooled at room temperature. A very recent review, proposed by Lavoine *et al.*, summarizes all the studies using such treatments before 2012 [25]. Their review provides a new interest with development of a pilot machine like the GEA system. Before that period, the Microfluidizer was mainly used at the lab scale.

(ii) The microfluidizer is equipment that also allows the defibrillation of cellulosic pulps (Figure 3). The fiber suspension is placed in an inlet reservoir, and then thanks to a pump intensifier generating high pressure, the slurry is accelerated and led into the interaction chamber.

When the pressurized product enters into the interaction chamber and passes through geometrically fixed micro-channels, very high velocities are achieved. Thin Z-shaped chambers with different sizes (400–200–100 µm) have to be used for the fibrillation process. The lower the chamber size, the higher the degree of fibrillation. At the end of the process, a heat exchanger cools down the product stream to ambient temperature. Lavoine *et al.* [25] give an overview of scientific works with such an apparatus, which have been largely performed in France and Scandinavia, where the first well-fibrillated NFCs were studied.

(iii) The grinder device (Figure 4), first proposed and developed by Masuko© (Tokyo, Japan), involves the breakdown of the cell wall structure thanks to the shearing forces generated by two grinding stones with countersense rotation. The pulp is passed between a static grinding stone and a rotating grinding stone revolving at about 1500 rpm. Lavoine *et al.* [25] sum-up also the different pre/post treatments applied to different cellulosic fibers to obtain NFC using a grinder device.

(iv) Other systems like cryo-crushing [18] refiners [26] or extruders [27] have been used based on the same idea. However up to know, they are not at all commonly used.

Without any pre-treatment, the number of passes of cellulose fibers into the fibrillation chamber is approximately 20, 10 and 5 to reach a good quality and a homogeneous fibrillation for Gaulin, GEA and Microfludizer apparatus respectively. The energy consumption is usually high and varies according to the devices used for the fibrillation. This is one of the main drawbacks related to the process of NFC production. Some pretreatments were developed by researchers in order to solve this problem and facilitate production at a larger-scale, as shown with a homogenizer.

### 2.3. Pre-Treatments

Nowadays, two main pretreatments can be applied to cellulose fibers to produce NFC, *i.e.*, (i) Enzymatic or (ii) TEMPO pretreatment.

(i) Enzymatic Pre-Treatment

Enzymatic pre-treatments enable NFC manufacture with significant reduced energy consumption. Inspired by nature, the idea is to limit interactions between microfibrils. Indeed, cellulose is degraded in nature by a set of enzymes called cellulases. They can be classified as A- and B- type cellulases, termed cellobiohydrolases, which are able to attack highly crystalline cellulose, or as C- and D-type cellulases or (endoglucanases) which generally require some disorder in the structure in order to degrade cellulose [28]. Cellobiohydrolases and endoglucanases can show also strong synergistic effects [29]. During preparation of NFC, isolated cellulases can be applied to modify the structure rather than degrading the cellulose. Some authors [14,29] found that endoglucanase pre-treatment facilitates disintegration of cellulosic wood fiber pulp into cellulosic nanofibers. Pretreated fibers subjected to the lowest enzyme concentration (0.02%) were successfully disintegrated while molecular weight and fiber length were well preserved. Between two refining steps, [14] performed an enzymatic treatment with endoglucanase before passing the pulp slurry through the microfluidizer which promotes cell wall delamination, and thus prevents the z-shaped chamber in the microfluidizer from blocking or clogging. They compared enzyme-pretreated NFC with non-pretreated NFC, as well as with a gentle and strong hydrolysis of pretreated NFC. Endoglucanase pre-treatment facilitates the disintegration of cellulosic wood fiber pulp by increasing its swelling in water. Moreover, this environmentally friendly pre-treatment confers a more favorable structure on the NFC, as it reduces the fiber length and increases the extent of fine material, compared to the result of acid hydrolysis pre-treatment. Their results showed that enzymatic pre-treatment gives much more homogeneous NFC suspensions. Thus, enzymatic pre-treatment is a very promising method for industrial applications and larger-scale NFC production. It is also one of the key steps in the first pilot production of NFC that was announced by Lindström’s group from Innventia (“Press Release: Nanocellulose—for the first time on a large scale—Innventia,” 2011). A quite recent study that was subdivided into two scientific papers [30,31] gave a more detailed analysis of the impact of enzymatic treatment on the final properties of the NFC obtained. In the experiments, two kinds of enzymes at different concentrations were tested as post- and pre-treatments. The results showed the importance of precisely detailing such post- or pre-treatment. Indeed depending on cellulase concentration, the morphology of ensuing NFC and their reinforcing effect in a matrix can be totally different. The authors insist on the need for precise manufacturing protocols when discussing NFC.

(ii) TEMPO Mediated Oxidation Pre-Treatment

Currently, the more commonly used chemical pre-treatment is TEMPO-mediated oxidation. Indeed, the TOCNs, or TEMPO-oxidized cellulose nanofibers, represent an entire category of nanocellulose worthy of consideration.

TEMPO-mediated oxidation is a well-known method for modifying selectively the surface of native cellulose under aqueous and mild conditions [9,10,32,33].

**Figure 5:**
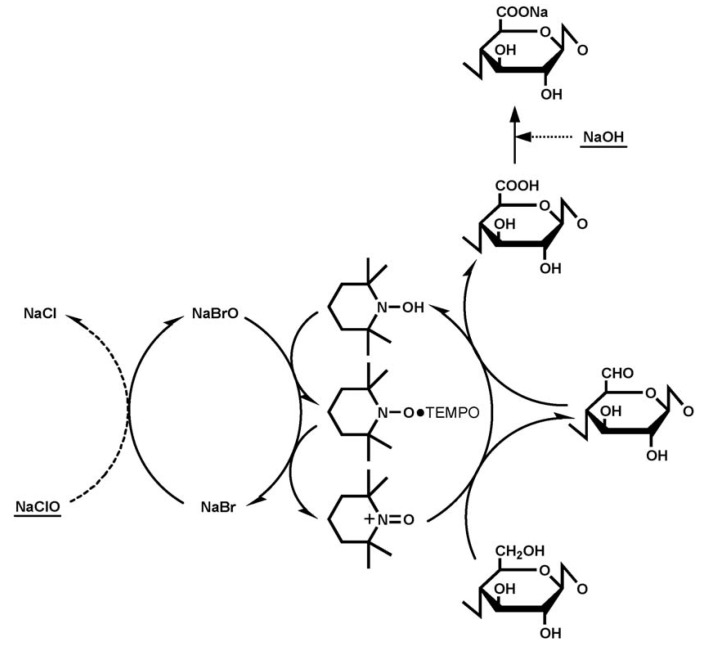
Schematic representation of the regioselective oxidation of cellulose by 2,2,6,6 tetramethyl-1-piperidinyloxy (TEMPO) process.

The basic principle of this form of pre-treatment consists of the oxidation of cellulose fibers via the addition of NaClO to aqueous cellulose suspensions in the presence of catalytic amounts of 2,2,6,6 tetramethyl-1-piperidinyloxy (TEMPO) and NaBr at pH 10–11 at room temperature (*cf*. Figure 5). The C6 primary hydroxyl groups of cellulose are thus selectively converted to carboxylate groups via the C6 aldehyde groups, and only NaClO and NaOH are consumed.

The higher the quantity of NaClO in the reaction medium, the higher is the number of carboxylic groups formed at the surface of the NFC and the stronger is the decrease in the degree of polymerization (DP). Isogai’s group [10,32] applied this treatment to many diverse sources: wood pulp, cotton linters, tunicate, bacterial cellulose, ramie, and spruce holocellulose *etc.* They defined the oxidation efficiency of their pre-treatment by the following equation [32]:

Oxidation efficiency (%) = 100 × {2 × (CT − CO) + (AT − AO)}/MNaClO(1)

where MNaClO is the quantity of NaClO added (mmol/g); CO and CT are the carboxylate contents (mmol/g) before and after oxidation, respectively, and AT and AO are the corresponding aldehyde contents (mmol/g), respectively.

Another TEMPO-mediated oxidation system has been reported with different conditions in comparison to the first one. This system operates at pH 7, NaClO replaces NaBr, and the primary oxidant is NaClO2 instead of NaClO.

Recently, a TEMPO electro-mediated reaction was also developed as an alternative method to oxidize the C6-primary hydroxyls of cellulose [34]. The authors applied two new systems to softwood bleached Kraft pulp: electro-mediated oxidation with TEMPO at pH 10, and 4-acetamido-TEMPO at pH 6.8 in a buffer solution. This new sustainable method could well replace the first two systems, although longer oxidation times are required. The yield is quite high (more than 80%). Moreover, it preserves the main characteristics of TEMPO-oxidized NFC produced from bleached softwood Kraft pulp [34] or from annual plant [35].

More details about TEMPO treatment, processes and application are summarized in a very recent review paper by Isogai [36].

Even if the washing step of these tempo-treated fibers is still the main drawback for their industrialization, they have several advantages. Indeed, compared to the energy consumption of repeated cycles of a high pressure homogenizer (700–1400 MJ/kg), TEMPO-mediated oxidation pre-treatment drastically decreases the consumption to values less than 7 MJ/kg. The nanofibrils within the fibers separate from each other more easily due to the repulsive forces of the ionized carboxylate groups, which overwhelm the hydrogen bonds holding them together [37]. TEMPO oxidation pre-treatment is usually followed by a mechanical treatment, which can be performed using the devices mentioned before but also with a simpler system like a mixer. However, it is worth keeping in mind that, one key step in this process is the “post-separation” of smaller NFC and bigger NFC by centrifugation. Only the supernatant (smaller NFC) is usually considered as TOCN. The ensuing TOCN material is shorter and thinner than with enzymatic pretreatment which presents other properties completely different from those obtained by the enzymatic way.

(iii) Other Pre-Treatments in the Literature

Other pre-treatments exist such as carboxymethylation [38] or acetylation [39] but they are less used. Lavoine *et al.* [25] summarized all pre- or post-treatments and devices used to obtain NFC. The workIt constitutes an exhaustive list of all sources used for the production of NFC. The publication deals with NFC barriers and gives an overview of all the grades available of NFC and the influence on barrier properties.

Cellulose surface modification, as briefly discussed in the introduction, has already been studied for several applications and several reviews have given a clear overview of the different existing strategies. However, none exists regarding NFC surface modification. The next section discusses the different strategies developed to physically or chemically modify NFCs.

## 3. NFC Surface Modifications Strategies

Due to the hydrophilic nature of cellulose, NFC cannot be uniformly dispersed in most non-polar polymer media and its suspension is a gel-like structure at very low concentration and the NFC forms films or aggregates once dried. Consequently, NFC modification is of interest in order to limit this phenomenum and open-up new applications. Compatibility with a wider variety of matrices used in coating colors or in extrusion can be attempted. NFC surface modification can also help to introduce new functionalities and to produce “active” NFC. In spite of the many methods already proposed for cellulose surface modification including a very recent review about functionalization of Cellulose Nanocrystals [40], reports on surface modification of nanocellulosic fibers are very limited in number.

**Figure 6:**
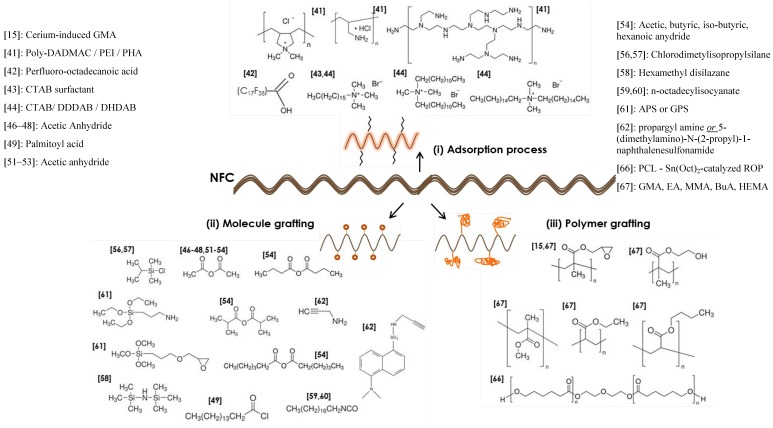
Exhaustive list of reagents used for modification of NFC. Three strategies adopted and described (**i**) physical adsorption, (**ii**) molecule surface modification and (**iii**) polymer grafting approaches reported in the literature (Sci Finder source updated in December 2012—the number in brackets refers to the reference number from which the information was taken. All reagents are identified by their IUPAC name).

The surface of cellulose nanoparticles can be modified and tuned either (i) by physical interactions or adsorption of molecules or macromolecules onto their surface or (ii) by using a chemical approach to achieve covalent bonds between cellulosic substrates and the grafting agent. Each strategy is detailed but as, a starting point, Figure 6 gives an exhaustive overview of all reagents used for physical adsorption, as well as molecules or polymers grafted at the surface of NFC to our knowledge.

Because of their nanoscale dimensions, nanofibrillated cellulose display a high surface area generally of the order of 50–70m^2^/g which greatly increase the quantity of surface hydroxyl groups available for surface modification and change the classic conditions of grafting. Moreover, the surface chemistry of NFC is primarily governed by its production procedure. Indeed, hydroxylated surfaces similar to native cellulose are classically obtained but as reported before, several strategies have been proposed to decrease the energy consumption for fibrillation. For example, TEMPO oxidation of cellulose introduces carboxylic acid groups at the surface of fibrillated cellulose. Carboxymethylation has also been used as pretreatment prior to mechanical defibrillation [41] and consequently also to modify the surface chemistry of the ensuing carboxymethylated NFC. Therefore, it is crucial to be precise about any NFC pretreatment when discussing NFC surface modification strategy. For example, the two latter NFCs are well adapted to the physical adsorption strategy first detailed below.

### 3.1. Surface Adsorption on NFC

The surface of cellulose nanoparticles can be tuned by using surfactants or polyelectrolyte adsorption. Surfactants are usually amphiphilic organic compounds, *i.e.*, compounds containing both hydrophobic groups (so-called tails) and hydrophilic groups (so-called heads). Cellulose films prepared from carboxymethylated NFC were modified by coating with various amounts of a fluorosurfactant, such as perfluorooctadecanoic acid (C_17_F_35_COOH) [42]. The authors demonstrated a strong decrease of dispersive surface energy after adsorption in comparison to carboxymethylated NFC, from 54.5 mN/m to 12 mN/m respectively.

The anionic surface of TEMPO-NFC can be easily modified with a cationic surfactant. As an example N-hexadecyl trimethylammonium bromide (also called cetyltrimethylammonium bromide CTAB) dissolved in water was deposited on the surface of NFC films [43,44]. The adsorbed layer of CTAB was found to increase the hydrophobicity of the film without affecting its mechanical properties significantly. CTAB, as well as didodecyl- (DDDAB) and dihexadecyl ammonium bromide (DHDAB) were used to control the water repellency of cellulose nanofibrils [44]. In this study, the surfactant was directly added to NFC in an aqueous suspension. Contact angle values were determined to be higher for TEMPO-NFC film dipped in CTAB solution in comparison to neat TEMPO-NFC film (60° and 42° respectively). The treated material was not fully hydrophobic but it was rendered more water repellent (lower adhesion with water). FE-SEM characterization was done on a covered filter paper with the mixture NFC-Tempo + CTAB.

Another way to modify surface properties of NFC is to use a polyelectrolyte solution. One of the most relevant scientific papers was published by Wägberg [41]. Indeed, they performed polyelectrolyte multilayer (PEM) using three different polyelectrolytes (Poly-DADMAC, PEI and PAH solutions). NFC used was obtained by carboxymethylation of the pulp and then homogenized. After titration the NFCs displayed a total charge of 515 µeq/g and assuming that all charges are located at the surface of the NFC, the equivalent quantity for adsorption of polyelectrolytes was then added. The combination of PEI and NFC in deionized water results in the formation of regular layers of NFC and PEI with layer thicknesses of 20 and 3 nm, respectively, after deposition of about 10 layers. By changing the salt concentration during adsorption of PDADMAC and PAH, it was possible to control the thickness of the PEM. The PEMs had different colors depending on the thickness of the multilayers and simple estimations of the thickness of the PEM from the colors, assuming dense cellulose layers, showed surprisingly good agreement with data from ellipsometry measurements. This indicates that the PEMs are basically compact films of cellulose with some cationic polyelectrolyte mixed/intercalated between the fibrils.

Very recently Martins *et al.* [45] proposed an innovative technique in order to produced nanopaper with antimicrobial activity using polyelectrolytes as binder between NFC and silver nanoparticles. This paper reports a Layer-by-Layer (L-b-L) assembly onto NFC with cationic polyelectrolytes (*i.e.*, PDDA, PHA and PEI) and anionic polyelectrolyte (*i.e.*, PSS). The adsorption of a first layer of cationic polyelectrolyte was performed on NFC, followed by a second layer deposited using PSS as anionic polyelectrolyte and finally recovered with a last layer of the same cationic polyelectrolyte. Then the Ag colloidal suspension was mixed with this modified NFC. This approach was successfully employed to impart antibacterial properties to NFC. The antibacterial activity was observed for NFC/Ag materials against different bacteria. The activity can be adjusted by varying the amount and characteristics of NFC/Ag used as nanofiller in the papers.

In conclusion, physical adsorption can be easily performed on charged NFCs to obtain more hydrophobic behavior. However, this procedure can induce some migrations phenomena of physically adsorbed moieties. That is why processes aimed at modifying NFCs chemically were developed.

### 3.2. Molecule Chemical Grafting

The hydrophobization of the cellulose surface has usually been achieved through the well-known cellulose esterification process, which basically uses carboxylic acid, acid anhydrides or acyl chlorides as reacting agents. Esterification is a reaction that introduces an ester functional group (O−C = O) onto the surface of cellulose by condensation of the previous reagents with a cellulosic alcohol group. Acetylation is the reaction that introduces an acetyl functional group CH_3_-C(=O)- onto the surface of cellulose. This basic reaction is also involved in the preparation of cellulose ester derivatives, such as the well-known cellulose acetate. The main target of this strategy is to keep the nanofibrillar structure so as to graft only the NFC surface.

A non-swelling media is classically used in this heterogeneous reaction mechanism in order to maintain the structure and properties. In this case, as the reaction only occurs on the cellulose chains located on the surface of the nanoparticles, the limitation on the extent of acetylation lies in the susceptibility and accessibility of the surface, which can produce several grades of modified materials with different degrees of substitution for example. In the research from Kim *et al.* [46], Bacterial cellulose (BC) was partially acetylated to modify its physical properties while preserving the microfibrillar morphology using anhydrous acetic acid and toluene as solvent reaction media (which are apolar, limiting the swelling of BC). In this case, the degree of acetyl substitution had a strong influence on material properties like stiffness and deformability of the acetylated BC. Mechanical properties of BC can be tuned depending on the degree of substitution. Thermal degradation resistance or optical properties have also been reported by Ifuku *et al.*, and Nogi *et al.* [47,48]. Under an oxidative atmosphere, the acetylated BC retains transparency properties after three hours at 200 °C in comparison to untreated BC films which lost their transparency after one hour at 200 °C. In addition to the acetylation methods, another process using a gas-phase method by evaporation of a large excess of palmitoyl chloride was investigated recently [49]. The method was developed for freeze-dried bacterial cellulose microfibrils dried by the critical point method. We have to bear in mind that this procedure to dry NFC induces some irreversible aggregates. The accessibility of hydroxyl groups at the surface can be reduced. The experimental conditions (160 °C for 4 h, 170 °C for 4/6/13 h, 180 °C for 4 h and 190 °C for 2 h), nature and conditioning of cellulose were found to be important factors controlling the extent of esterification and morphology of the grafted nanoparticles. In addition, it was observed that the esterification proceeded from the surface of the cellulosic substrate to the crystalline core. This feature was confirmed by SEM analyses, which show clearly an increase in the diameter of the microfibrils and penetration depth of the chemical modification. Based on the same idea, Rodionova *et al.* [50] used a gas-phase esterification on NFC films with trifluoroacetic acid anhydride (TFAA) and acetic acid (AcOH) with several ratios (1:2 and 2:1) at 22 °C and 40 °C for 30 min or 40 min. Main results show an increase in the contact angle value (41.2° for unmodified film and 71.2° for esterified films). This gas phase esterification seems to be an effective technique for surface modification of NFC films or NFC aggregates. However, not all OH groups are available in this case and consequently, only a slight influence is observed.

Only few studies, dealing with the esterification of the NFCs surface can be found in the literature. Tingaut *et al.* [51], used a heterogeneous catalytic method thanks to a solvent exchange of a NFC suspension from water to DMF. The final product displayed several grades of acetyl content from 1.5% to 17% of acetylated groups. Such grafted NFCs are then used in nanocomposite applications and enhance interface adhesion with matrix like poly(lactic acid) through a solvent casting approach in chloroform. The authors showed that NFC with increasing percentage of acetylation (%Ac) provided more translucent nanocomposites with reduced hygroscopicity and improved thermal stability in comparison to unmodified NFC. All these properties could be fine-tuned through an accurate control of Ac%.

A second study concerned acetylation onto bleached cellulosic fibers [52] before mechanical disintegration. This facilitated the production of NFCs with high DS (1.07) and contact angle value (114°). It means that the fibers and thus the nanofibers are strongly modified and not only at the surface due to the decrease of the crystallinity index of the nanofibers from 81.2 to 74%. Rodionova *et al.* [53] used acetylation to increase the barrier property of NFC films with a contact angle value around 82° obtained for a DS of 0.7 for 1h of reaction. The main results of these acetylated NFC films showed no significant changes to the mechanical properties but the oxygen transmission rate was comparable to those of common packaging materials.

A novel method for chemical surface esterification of NFC in order to impart hydrophobic properties by using solvent exchange in ionic liquids was recently developed by Missoum *et al.*, in 2012 [54] using anhydrides. Results obtained proved that the chemical surface modification occurred only at the surface of the NFC. This characterization was possible thanks to the use of a powerful technique SIMS (Secondary Ion Mass Spectrometry).

**Figure 7:**
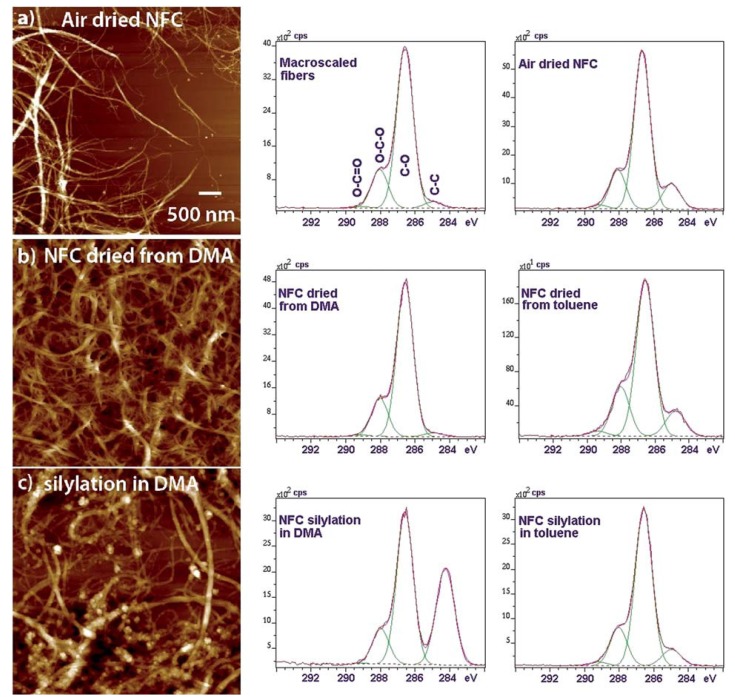
AFM and XPS data for neat and silylated NFCs, in DMA and toluene. Principle of “surface adaptation” (Reproduced from Johansson *et al.* 2011 [58]. Copyright permission of the Royal Society of Chemistry).

Similarly to acetyl chloride, chlorosilane has been used for NFC modification. Silylation consists of the introduction of substituted silyl groups R3Si onto the surface of cellulose nanoparticles. Goussé *et al.* [55] utilized isopropyl dimethylchlorosilane in toluene (after solvent exchange steps) for surface silylation of cellulose nanofibrils. These authors claimed that nanofibrils retained their morphology under mild silylation conditions and could be dispersed in a non-flocculating manner into organic solvents. Andresen *et al.* [56] hydrophobized NFC via partial surface silylation using the same silylation agent and reported that when silylation conditions were too harsh, partial solubilization of NFC and loss of nanostructure could occur. Films prepared from modified cellulose by solution casting showed a very high water contact angle (117–146°). It is probable that in addition to the decreased surface energy, the higher surface roughness (as a result of less hydrogen bonding) could contribute to increased hydrophobicity. It was also reported that such hydrophobized NFCs could be used for the stabilization of water-in-oil type emulsions [57]. More recently, Johansson *et al.* [58] demonstrated the influence of solvent exchange on NFC sylilation. DMA and toluene were used as solvent for the chemical surface modification. It seems that cellulose surface adaptation can be carried out depending on the solvent used in order to minimize its free surface energy. The free, accessible hydroxyl groups generate the high surface free energy of the cellulose surface. This very recent paper launched a discussion about OH accessibility at the surface of the NFC depending on the solvent process (Figure 7).

To avoid HCl or carboxylic acid by-products obtained during these esterifications and sylilations, some carbanilations were also recently proposed. Cellulose nanofibrils extracted from sisal fibers were chemically modified with n-octadecyl isocyanate (C_18_H_37_NCO) [59]. The surface chemical modification was carried out in toluene using for the first time an *in-situ* solvent exchange procedure to avoid NFC aggregation observed previously. Never-dried NFC was grafted after solvent exchange to acetone and then to dry toluene.

Based on the same procedure, a study published by Missoum *et al.*, (2012) [60] was performed in order to check the influence of different grafting agent quantities on the surface organization of grafted moieties and the properties of the ensuing modified NFC.

Recent strategies in aqueous media have been developed for cellulose grafting but again very few deal with NFC grafting. We can however note the use of silane and click chemistry techniques. For example, hydrophobization of NFC was also obtained by grafting 3-aminopropyltriethoxysilane (APS) and 3-glycidoxypropyltrimethoxysilane (GPS) [61]. NFC and coupling agents were mixed in acetone, and the mixture was filtered and dried. After treatment, better and stronger adhesion between NFC and the epoxy polymer used as the matrix was observed, which resulted in better mechanical properties of the composite materials. Click chemistry is tailored to generate substances by joining small units together under mild conditions. One of the most popular reactions within the click chemistry concept is the azide alkyne Huisgen cyclo-addition using a copper catalyst at room temperature. First, reactive azide groups were introduced onto the surface of NFC by the etherification of 1-azido-2,3-poxypropane in alkaline water/isopropanol-mixture at ambient temperature [62]. Then the azide groups were reacted with propargyl amine utilizing copper catalyzed azide-alkyne cycloaddition (CuAAC), leading to a pH-responsive 1,2,3-triazole-4-methanamine decorated NFC. Very recently, based on the same technique, Filpponen *et al.* [63] developed a generic and versatile method based on click chemistry for grafting all cellulosic substrates. They demonstrated that cellulose can be modified by exploiting the natural tendency of CMC to physically adsorb onto cellulose in aqueous medium, even after azide or alkyne functionalization of CMC. This property combined with a subsequent click chemistry reaction enabled modification of the cellulosic surfaces. Several cellulosic substrates (amorphous and nanofibrillar cellulose films, filter paper) as well as versatile modifications (protein, fluorescent labeling, and PEG grafting) were performed. This method has a potential to set an altogether alternative trend for heterogeneous modification of cellulose. The last strategy used is polymer grafting onto NFC.

### 3.3. Polymer Grafting

Surface chemical modification of cellulose nanoparticles can be achieved by covalently attaching small molecules, as well as polymers. The general objective of this chemical modification is to increase the apolar character of the nanoparticle and have a better compatibility with hydrophobic polymer matrices. Two main approaches can be used to graft polymers onto surfaces, *i.e.*, “grafting onto” or “grafting from”. The first method was extensively used for the fibers or the NCC particles and not for the NFC. The “grafting onto” approach consists of: (i) mixing the cellulosic nanoparticles with an existing polymer and a coupling agent to attach the polymer to the nanoparticle surface; or (ii) activating the cellulose substrates (or the polymer) and grafting one (or the other) onto the other one. In this approach, one cannot expect high grafting densities because of steric hindrance induced by polymeric chains. Moreover, the viscosity of the reaction medium is usually high because of the presence of macromolecular chains. However, its main advantage is that the properties of the resulting material are perfectly controlled since the molecular weight of the attached polymer can be characterized before grafting.

The second strategy, *i.e.*, “grafting from” approach, consists of mixing the cellulosic nanoparticles or the activated cellulosic nanoparticles with a monomer and an initiator agent to induce polymerization of the monomer from the nanoparticle surface. Because of the lower viscosity of the medium and the limitation of steric hindrance, this strategy has proven to be a very effective way to create high grafting densities on the surface. However, it is difficult to control and determine precisely the molecular weight of the grafted polymer, which is usually limited to a low degree of polymerization. The quantity of homopolymer (non-grafted) is also not so easy to determine. Several studies reported the preparation of PCL-grafted cellulose nanoparticles using the “grafting from” strategy. PCL is traditionally prepared by the Sn(Oct)_2_-catalyzed ring-opening polymerization (ROP) of cyclicɛ-caprolactone monomer. This approach was used to prepare PCL-grafted ramie [64] and native linter [65] cellulose nanocrystals. A similar approach was used to graft PCL onto the surface of NFC.

Freeze-dried NFC was mixed with ɛ-CL monomer and a grafting reaction was conducted with a catalytic amount of Sn(Oct)_2_ at 95 °C for 18–20 h [66]. By changing the amount of added free initiator to monomer, the amount of PCL on the NFC surface was altered to optimize the graft length. Different theoretical lengths of the PCL chains, *i.e.*, DP 300, 600 and 1200, were investigated. The experimental molecular weights of free PCL formed during the grafting reaction were estimated from NMR and size exclusion chromatography (SEC). As expected, the obtained values were significantly lower than the theoretical ones since the theoretical molecular weight was calculated from the ratio of added monomer to free initiator, whereas the experimental value depends on the added monomer to free initiator, as well as the number of initiating groups on the NFC surface. TGA was used to estimate the composition of PCL-grafted NFC. PCL contents of 16%, 19% and 21% were reported depending on the amount of free initiator in the system. Crystallization of grafted PCL was observed, but because of the lower mobility of these chains compared to free PCL, a lower melting point and degree of crystallinity, as well as a longer crystallization time were reported.

Another possibility of “grafting from” is to use classical radical polymerization thanks to a redox initiated free radical system such as Cerium Ammonium Nitrate (CAN). The cerium (IV) ion is a powerful oxidant agent for an alcohol containing 1,2-glycol groups. The mechanism of ceric ion reaction involves the formation of a chelate complex that decomposes to generate free radicals on the cellulose backbone. Epoxy functionality was introduced onto the surface of NFC by oxidation followed by radical polymerization by Cerium (IV) of glycidyl methacrylate (GMA) [15]. Significant degradation of the cellulose chains occurred because of the formation of radicals in the reaction involving ammonium cerium nitrate. However, it was shown that the treatment resulted in only a slight reduction in the molecular weight of cellulose. In the same study, Stenstad *et al.* [15] demonstrated that the coupling of NFC with maleic anhydride introduced vinyl groups that could be used as a starting point for grafting reactions for monomers that are insoluble in water, as an alternative to the cerium-induced grafting method. NFC was also grafted in aqueous solution using a redox-initiated free radical polymerization with two acrylates and three methacrylates [67]. Cerium ammonium nitrate was also used as initiator. The graft copolymerization was dominant over homopolymerization for all monomers. The highest graft yield was obtained with butyl acrylate (BuA) and glycidyl methacrylate (GMA) with 80 wt%. According to AFM imaging, the nanofibrillar structure of the cellulose was preserved during synthesis, which means that the polymeric modification occurred without significant nanofibril aggregation. Another drawback of this grafting strategy is the high amount of homopolymer formed and the difficulty to distinguish it from the grafted polymer.

Table 1 collates the results of chemical surface modifications onto NFC. All of them are very recent and half of them have been published over the four last years. Indeed, NFC production has clearly evolved during the last three years.

**Table 1:** All physical and chemical strategies used to impart grafting onto Nanofibrillated Cellulose (NFC).

Source of cellulose	Pretreatment		Reagent		Solvent/Process	DS *		Reference	
**Surface adsorption**									
Sulfite softwood dissolving pulp	Carboxy-methylation	Poly-DADMAC/ PEI/PHA			LbL assembly	Surf.Charge 515 µeq/g		[41]	
Sulfite softwood dissolving pulp	Carboxy-methylation	Perfluoro-octadecanoic acid			Coating on films	nd.		[42]	
Softwood bleached Kraft pulp	TEMPO oxidation	CTAB surfactant			Coating on films	nd.		[43]	
Softwood bleached Kraft pulp	TEMPO oxidation	CTAB/DDDAB/DHDAB surfactant			Mixing	0.08–0.27		[44]	
**Molecule chemical grafting**									
Bacterial Cellulose	*Acetobacter xylinum*	Acetic anhydride			Acetic acid + toluene	0.04–2.77		[46]	
Bacterial Cellulose	nc.	Acetic anhydride			No solvent	nd.		[48]	
Bacterial Cellulose	*Acetobacter xylinum*	Acetic anhydride			Acetic acid + toluene	0.15–1.76		[47]	
Bacterial Cellulose	*Nata de coco*	Palmitoyl acid			Gas phase	1.47–2.01		[49]	
Bleached sulphite wood pulp	nc. (Supplied by Borregaard)	Acetic anhydride			DMF	%Ac. 1.5–17		[51]	
Kenaf Bast Fibers	Acetylation	Acetic anhydride			Pyridine	1.07		[52]	
Norway Spruce Kraft Pulp	No	Acetic anhydride			Toluene	0.56–0.91		[53]	
Sweden Domsjö Pulp	Enzyme	Acetic/Butyric/ Iso-butyric/Hexanoic anhydride			bmimPF_6_ Ionic liquid	0.3/0.3/0.2/0.3		[54]	
Sugar Beet Pulp	No	Isopropyl dimethylchlorosilane			Toluene	DSS = 0.025–0.36		[55]	
Bleached Spruce Sulfite Cellulose	nc. (Supplied by Borregaard)	Chlorodimethyl isopropylsilane			Toluene	DSS = 0–0.16		[56]	
Bleached Spruce Sulfite Cellulose	nc. (Supplied by Borregaard)	Chlorodimethyl isopropylsilane			Methanol water	n.c		[57]	
Kraft Pulp	nc. (Supplied by Daicel)	APS or GPS			Acetone	n.c		[61]	
Bleached Spruce Sulfite Cellulose	n.c	Hexamethyl disilazane			DMA or Toluene	n.c		[58]	
Bleached Sisal fibers	n.c	n-octadecyl isocyanate			Toluene	0.09		[59]	
Bleached Eucalyptus fibers	Enzyme	n-octadecyl isocyanate			Toluene	0.09		[60]	
**Molecule chemical grafting**									
Bleached Birch pulp	nc. (Supplied by Finnish center)			propargyl amine *or* 5-(dimethylamino)-N-(2-propyl)-1-naphtha lenesulfonamide	Water	0.013 0.014		[62]	
**Polymer grafting**									
Bleached Spruce Sulfite Cellulose	n.c			Cerium-induced GMA	Water + HNO_3_	n.c		[15]	
Bleached Birch Pulp	n.c			Cerium-induced GMA EA MMA BuA, HEMA	Water + HNO_3_	Graft yield 96%–99% 81%–85% 56%–75% 86%–89% 18%–63%		[67]	
Bleached sulfite softwood dissolving pulp (Domsjö)	Carboxy-methylation (DS = 0.089)			PCL-Sn(Oct)_2_-catalyzed ROP	Toluene	16%–19%–21%		[66]	

Regardless of all the grafting strategies mentioned before, the characterization of the ensuing materials is a key step in the comprehension of their properties. The use of nanoscale materials requires a precise and complete characterization. Therefore, techniques are becoming more varied and push-ups to achieve the required standards in the reliability of results are obtained. This is especially true and important in the present report due to the method adopted for the modification of NFC based on a surface grafting of this nanobiomaterial.

## 4. Conclusions

Cellulose is one of the most fascinating natural polymers. It is also a renewable material largely produced by photosynthesis. It can be considered at different levels with its multi-level organization and hierarchical structure. One of its promising derivatives is certainly Nanofibrillated cellulose. As described, it can be used in several applications (such as in papers or nanocomposites) with substantially enhanced properties. Nevertheless, some drawbacks limit its use, such as aggregation, low concentration suspension and compatibility with hydrophobic polymeric matrices for instance.

One solution to overcome these problems is chemical modification of NFC. Furthermore, such an operation could be the occasion to provide new functionalities to these NFCs. The surface modification of NFC is very innovative with less than 30 papers worldwide. To date mainly solvent based or toxic systems have been studied, which limit the extension of these finding to industrial scale-up. This is the main reason why some European projects such as the SUNPAP project has been launched to avoid the use of such solvents. More and more researchers are focusing on the development of greener strategies regarding chemical surface modification, which can certainly be considered as the main perspective for NFC functionalization.
